# Sciatic Nerve Bifurcation and Anatomical Reference Points: Diagnostic and Clinical Implications

**DOI:** 10.3390/diagnostics16162522

**Published:** 2026-08-10

**Authors:** Mehmet Aydin, Cemre Savaşan, Mehmet Yilmaz, Ege Ozkubat, Gokcen Emmez, Alp Bayramoglu, Tulin Sen Esmer

**Affiliations:** 1 Department of Anatomy, Hamidiye International School of Medicine, University of Health Sciences, Istanbul 34668, Turkey; drmehmetaydin@yahoo.com; 2 Department of Anatomy, School of Medicine, Acibadem Mehmet Ali Aydinlar University, Istanbul 34752, Turkey; alp.bayramoglu@acibadem.edu.tr; 3 Department of Anatomy, School of Medicine, Ankara University, Ankara 06230, Turkey; md.mehmetyilmaz@gmail.com (M.Y.); tulin_sen@yahoo.com (T.S.E.); 4 Department of Anaesthesiology and Reanimation, Faculty of Medicine, Gazi University, Ankara 06500, Turkey; egeozkubat@gmail.com (E.O.); gokcenemmez@gmail.com (G.E.)

**Keywords:** sciatic nerve, bifurcation, tibial nerve, common fibular nerve, posterior intercondylar reference line, Beaton and Anson classification, ultrasonography, popliteal sciatic nerve block

## Abstract

**Background/Objectives:** The level of the sciatic nerve bifurcation is clinically relevant for diagnostic ultrasonography, ultrasound-guided popliteal sciatic nerve block, posterior-thigh interventions, and surgical approaches to the hip, thigh, and popliteal region. Although the sciatic nerve is classically described as dividing near the popliteal fossa, its terminal branching level may vary considerably. This study aimed to evaluate the level of sciatic nerve bifurcation and its morphometric relationship to clinically relevant anatomical landmarks, with particular emphasis on the posterior intercondylar reference line. **Methods:** This study was conducted on 56 formalin-fixed adult human lower-extremity specimens. The bifurcation level was categorized as femoral-level, gluteal-level, or separate emergence of the tibial and common fibular components. The sciatic nerve–piriformis relationship was assessed according to the Beaton and Anson classification. Morphometric measurements were obtained from the bifurcation point to the greater trochanter, ischial tuberosity, and posterior intercondylar reference line. **Results:** Femoral-level bifurcation was observed in 51 of 56 specimens (91.1%), gluteal-level bifurcation in 1 specimen (1.8%), and separate emergence in 4 specimens (7.1%). Type A was the predominant Beaton and Anson pattern, observed in 50 specimens (89.3%). Type B and Type D were each observed in 2 specimens (3.6%), and 2 specimens (3.6%) demonstrated an atypical superior gemellus-related course. All measurable bifurcation points were located proximal to the posterior intercondylar reference line. Among femoral-level bifurcations, the mean distance from this line to the bifurcation point was 8.67 ± 4.89 cm. No statistically significant sex- or side-based differences were observed in the available morphometric parameters. **Conclusions:** Sciatic nerve bifurcation most commonly occurred at the femoral level, but clinically relevant variations were observed. The posterior intercondylar reference line may provide a useful distal femoral orientation landmark for posterior knee and distal thigh assessment. However, it should complement, rather than replace, direct ultrasonographic visualization of the sciatic nerve and its terminal branches.

## 1. Introduction

The sciatic nerve, the largest peripheral nerve in the human body, plays a critical role in motor and sensory innervation of the lower extremity. Because of its extensive course and close relationship to the osseous and muscular structures of the gluteal and posterior thigh regions, the sciatic nerve is frequently encountered during orthopedic surgery, regional anesthesia, pain management procedures, and imaging-guided interventions. Variations in sciatic nerve anatomy and bifurcation patterns are therefore clinically significant because they may affect procedural success and increase the risk of iatrogenic nerve injury [1,2,3]. In addition, high-resolution ultrasonography has increasingly enabled direct visualization of the sciatic nerve and its terminal branches, further underscoring the diagnostic value of detailed anatomical knowledge [4,5].

The level of sciatic nerve bifurcation shows substantial anatomical variability among individuals. Although the sciatic nerve typically divides into the tibial and common fibular nerves near the popliteal region, bifurcation may occur more proximally at varying levels of the posterior thigh [6]. Such variations may have important clinical consequences, particularly during popliteal sciatic nerve blocks and other lower-extremity interventions. Variability in the level of sciatic nerve bifurcation has been suggested to contribute to incomplete popliteal nerve blockade because local anesthetic deposited distal to the bifurcation may affect only one terminal component. Conversely, during selective tibial nerve blockade, spread toward the common fibular component may produce unintended motor involvement [7,8]. In addition, inadequate awareness of sciatic nerve variations may increase the risk of iatrogenic injury during surgical procedures in the gluteal and posterior thigh regions [1].

Despite the growing use of ultrasound-guided techniques in regional anesthesia and interventional procedures, landmark-based approaches remain important in daily clinical practice. Anatomical reference points, including the greater trochanter, ischial tuberosity, distal femoral landmarks, and popliteal-region references, are often used to estimate the course and bifurcation level of the sciatic nerve. Ultrasound-guided approaches also rely on recognizable anatomical relationships; for example, the sciatic nerve can be identified between the ischial tuberosity and the greater trochanter at the subgluteal level and traced distally to its bifurcation in the popliteal region [9]. Nevertheless, considerable individual variability may limit the reliability of these reference points. Inadequate anatomical orientation may lead to incomplete nerve blockade, prolonged procedural time, multiple needle attempts, or inadvertent neural injury. Furthermore, landmark-guided approaches remain particularly relevant in settings with limited access to imaging modalities and in patients with challenging sonographic anatomy. Therefore, detailed morphometric assessment of the relationship between the sciatic nerve and clinically relevant anatomical landmarks may provide valuable guidance for surgical and anesthetic interventions [2,7,8].

Although numerous studies have investigated anatomical variations in the sciatic nerve, most have focused on its relationship with the piriformis muscle, the prevalence of high bifurcation patterns, or isolated popliteal reference points. Moreover, classifications centered on the piriformis muscle may not fully capture relationships with the short external rotator muscles of the deep gluteal region that are independent of the piriformis. However, there remains a need for morphometric studies that evaluate the sciatic nerve bifurcation level relative to multiple clinically relevant anatomical reference points along the posterior thigh. Previous studies have examined individual reference points such as the popliteal crease or distal femoral landmarks; however, further anatomical characterization is still required to integrate proximal and distal reference points, including the greater trochanter, ischial tuberosity, and posterior intercondylar reference line, into a single anatomical assessment [9,10,11]. A more detailed understanding of these anatomical relationships may improve anatomical orientation during diagnostic imaging, regional anesthesia, posterior thigh interventions, and surgical procedures involving the sciatic nerve.

Therefore, the present study aimed to determine the level of the sciatic nerve bifurcation and to investigate its morphometric relationship with clinically relevant anatomical reference points in the posterior thigh and popliteal region. In addition, the study assessed the distribution of bifurcation patterns and their association with lower-extremity morphometric parameters. The novelty of the present study lies in the simultaneous morphometric evaluation of the sciatic nerve bifurcation relative to the greater trochanter, ischial tuberosity, and posterior intercondylar reference line. Integrating these landmarks within a single proximal-to-distal framework provides a detailed anatomical characterization of the bifurcation level, with particular emphasis on the posterior intercondylar reference line. The findings of this study may contribute to improved anatomical orientation during diagnostic imaging, regional anesthesia, posterior thigh interventions, and surgical procedures involving the sciatic nerve.

## 2. Materials and Methods

### 2.1. Study Design and Specimens

This descriptive anatomical study was conducted on 56 formalin-fixed adult human lower-extremity specimens from the Department of Anatomy’s anatomical specimen collection at Ankara University School of Medicine. The study was designed to evaluate the level of sciatic nerve bifurcation and its morphometric relationship to clinically relevant anatomical landmarks in the gluteal, posterior thigh, and popliteal regions.

The sample comprised 31 right-sided and 25 left-sided lower extremities, of which 38 were male-derived and 18 were female-derived. The anatomical collection included both bilateral pairs and isolated unilateral specimens; therefore, a contralateral limb was not available in every case, accounting for the unequal distribution of right and left sides. Sex and side were recorded for each specimen before anatomical evaluation.

According to the institutional review document, the study was deemed exempt and did not require formal approval from the Institutional Review Board because it involved anonymized formalin-fixed adult human anatomical specimens and no identifiable human data. All methods were conducted in accordance with relevant institutional guidelines and regulations. Informed consent for the use of donated anatomical material for educational and scientific research purposes had been obtained from all donors at the time of donation, in accordance with institutional body donation procedures.

Specimens with evidence of major lower-extremity trauma, prior surgical intervention, severe deformity, gross anatomical disruption, or marked damage to the gluteal, posterior thigh, or popliteal regions that precluded reliable identification of the sciatic nerve or relevant anatomical landmarks were excluded from the corresponding morphometric analyses. Because certain anatomical landmarks were not preserved or sufficiently exposed in all specimens, morphometric analyses were based on the number of valid observations available for each parameter.

### 2.2. Dissection Procedure

**Figure 1:**
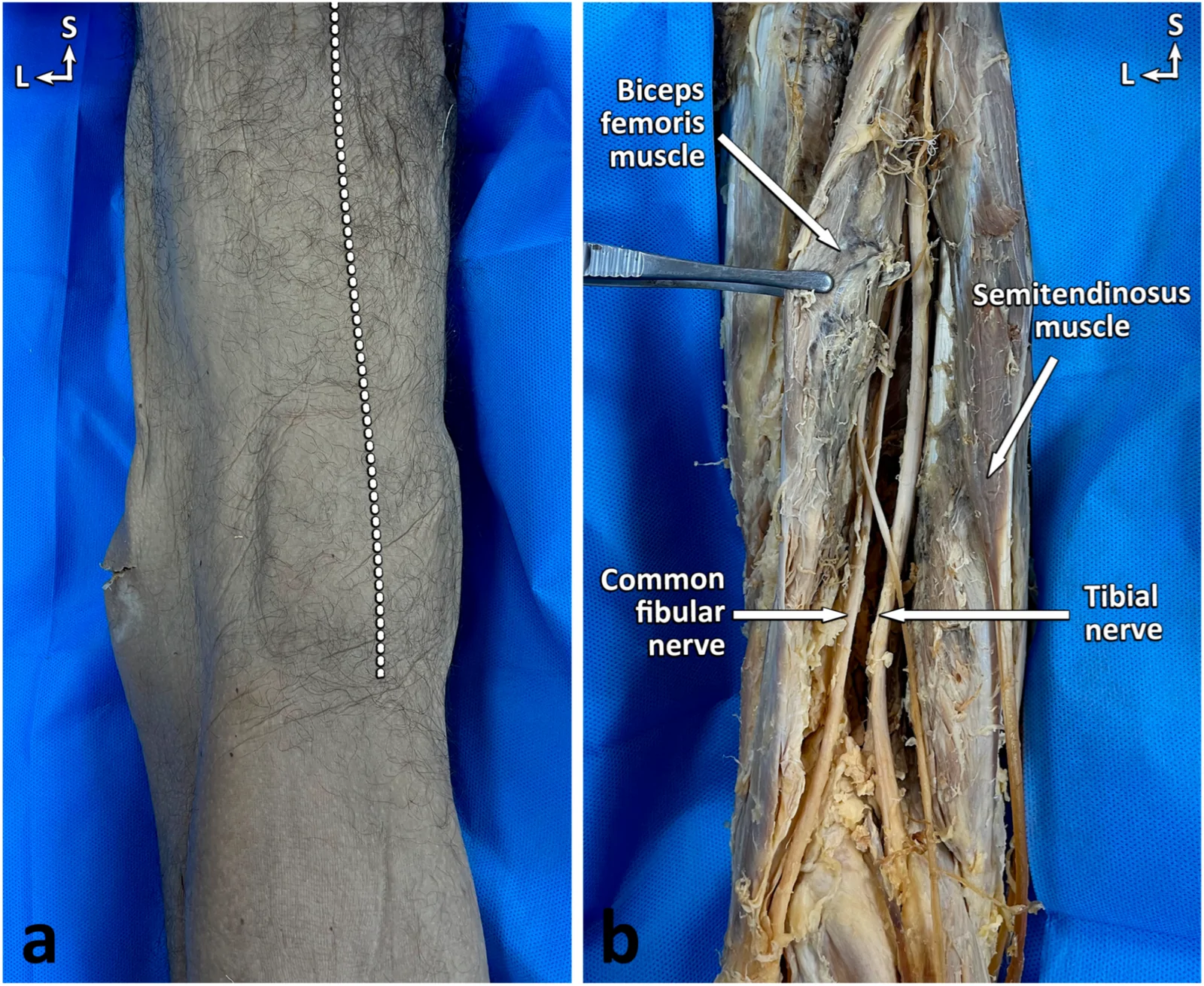
Representative posterior thigh dissection approach and exposure of the terminal components of the sciatic nerve. (**a**) Posterior view of the left lower extremity before dissection. The dashed line indicates the planned longitudinal skin incision to access the posterior thigh compartment. (**b**) Dissected posterior thigh showing the relationship of the terminal components of the sciatic nerve to the surrounding hamstring muscles. The common fibular nerve is lateral, whereas the tibial nerve is medial, between the biceps femoris and semitendinosus muscles. S: superior; L: lateral.

All dissections were performed with specimens in the prone position (Figure 1a). The skin, superficial fascia, and deep fascia of the gluteal region, posterior thigh, and popliteal fossa were carefully removed. The gluteus maximus muscle was reflected as needed to expose the proximal course of the sciatic nerve. The sciatic nerve was then traced distally along the posterior thigh until its division into the tibial and common fibular nerves was identified (Figure 1b).

The sciatic nerve bifurcation point was defined as the first anatomical point at which the tibial and common fibular components separated and continued as distinct neural structures. In specimens in which the tibial and common fibular components emerged separately without forming a typical sciatic nerve trunk, this pattern was recorded as separate emergence. These specimens were included in the anatomical variation analysis but were excluded from morphometric measurements that require a single point of sciatic nerve bifurcation.

The level of sciatic nerve bifurcation was categorized based on its relationship to the inferior gluteal fold. Bifurcations located proximal to the inferior gluteal fold were classified as gluteal-level bifurcations, whereas those located distal to the inferior gluteal fold were classified as femoral-level bifurcations. Specimens with separate emergence of the tibial and common fibular components were classified separately.

### 2.3. Morphometric Measurements

After exposure of the sciatic nerve and identification of the bifurcation point, morphometric measurements were obtained using clinically relevant anatomical landmarks in the gluteal, posterior thigh, and popliteal regions. All measurements were straight-line distances from the sciatic nerve bifurcation point to predefined anatomical reference levels.

The relationship between the sciatic nerve and the piriformis muscle was evaluated according to the Beaton and Anson classification [12]. Type A represents an undivided sciatic nerve passing inferior to the piriformis muscle; Type B, the common fibular component passing through the piriformis and the tibial component passing inferior to it; Type C, the common fibular component passing superior to the piriformis and the tibial component passing inferior to it; Type D, an undivided sciatic nerve passing through the piriformis; Type E, the common fibular component passing superior to the piriformis and the tibial component passing through it; and Type F, an undivided sciatic nerve passing superior to the piriformis. Anatomical relationships that could not be assigned to these six classical types were documented separately.

The greater trochanter was identified as the most prominent lateral bony point of the proximal femur. The ischial tuberosity was identified as the most prominent bony prominence of the ischium. The posterior intercondylar reference line was defined as a transverse line connecting the posterior aspects of the medial and lateral femoral condyles. The anterior superior iliac spine–lateral femoral condyle (ASIS–LFC) distance was recorded as the straight-line distance between these two landmarks. These readily identifiable and clinically palpable bony landmarks were selected to provide a standardized external dimension for normalization.

Distances from the sciatic nerve bifurcation point to the greater trochanter reference level, ischial tuberosity reference level, and the posterior intercondylar reference line were measured. For the greater trochanter and ischial tuberosity measurements, distances were recorded as signed values based on the position of the bifurcation point relative to the corresponding reference level. Negative values indicated that the bifurcation point was proximal to the reference level, whereas positive values indicated that it was distal to the reference level. Distances from the posterior intercondylar reference line were recorded as proximal distances from the reference line to the bifurcation point.

To account for differences in specimen size, normalized morphometric ratios were calculated when both the ASIS–LFC distance and the corresponding landmark-to-bifurcation distance were available. The ratios were computed using the following formula:


Normalized ratio = landmark-to-bifurcation distance/ASIS–LFC distance × 100

All measurements were performed using a standardized protocol, with specimens maintained in the prone position. Each morphometric parameter was measured once by two observers working together. Both observers verified the anatomical landmarks, instrument placement, and measurement reading, and only the value agreed upon by both observers was recorded. Measurements within the measuring range of the digital caliper were obtained using a digital caliper with a sensitivity of 0.01 mm (Mitutoyo Corporation, Kanagawa, Japan), whereas distances exceeding this range were measured using a measuring tape. All measurements were recorded in centimeters. Morphometric measurements were obtained only when the required anatomical landmark and, where applicable, a single sciatic nerve bifurcation point could be reliably identified. When a landmark was insufficiently preserved to permit reliable identification, the corresponding measurement was recorded as unavailable. Specimens showing separate emergence of the tibial and common fibular components lacked a single sciatic nerve bifurcation point and were excluded only from measurements requiring such a point. The anatomical reference points, measurement definitions, and interpretation of the recorded distances are summarized in Table 1.

### 2.4. Statistical Analysis

Statistical analyses were conducted using Python version 3.11 and standard scientific computing libraries, as well as Microsoft Excel (Microsoft Corp., Redmond, WA, USA). Categorical variables were summarized as frequencies and percentages. Continuous variables were summarized as mean ± standard deviation, median, minimum–maximum range, and interquartile range, as appropriate.

Morphometric analyses were based on available valid observations for each parameter. Missing morphometric values were not imputed. Specimens with separate emergence of the tibial and common fibular components were included in the anatomical variation analysis but excluded from measurements requiring a typical sciatic nerve bifurcation point.

The distribution of continuous variables was assessed using the Shapiro–Wilk test and visual inspection of distribution plots. Sex- and side-based comparisons were performed for morphometric parameters with sufficient valid observations. Because complete donor-matched right–left pairs were not available for the entire sample, no within-donor paired comparison was performed; side-based comparisons were conducted at the individual lower-extremity level. As the assumption of normality was not consistently met across the morphometric parameters and their sex- and side-based subgroups, all sex- and side-based morphometric comparisons were performed using the Mann–Whitney U test. Categorical variables, including bifurcation category and Beaton and Anson pattern, were compared descriptively; Fisher’s exact test was used when appropriate because of low expected cell counts in some categories.

A *p*-value of <0.05 was considered statistically significant.

## 3. Results

### 3.1. Specimen Characteristics and Bifurcation-Level Distribution

A total of 56 adult lower extremities were examined in the present study. The general specimen characteristics and bifurcation-level distribution are summarized in Table 2.

**Figure 2:**
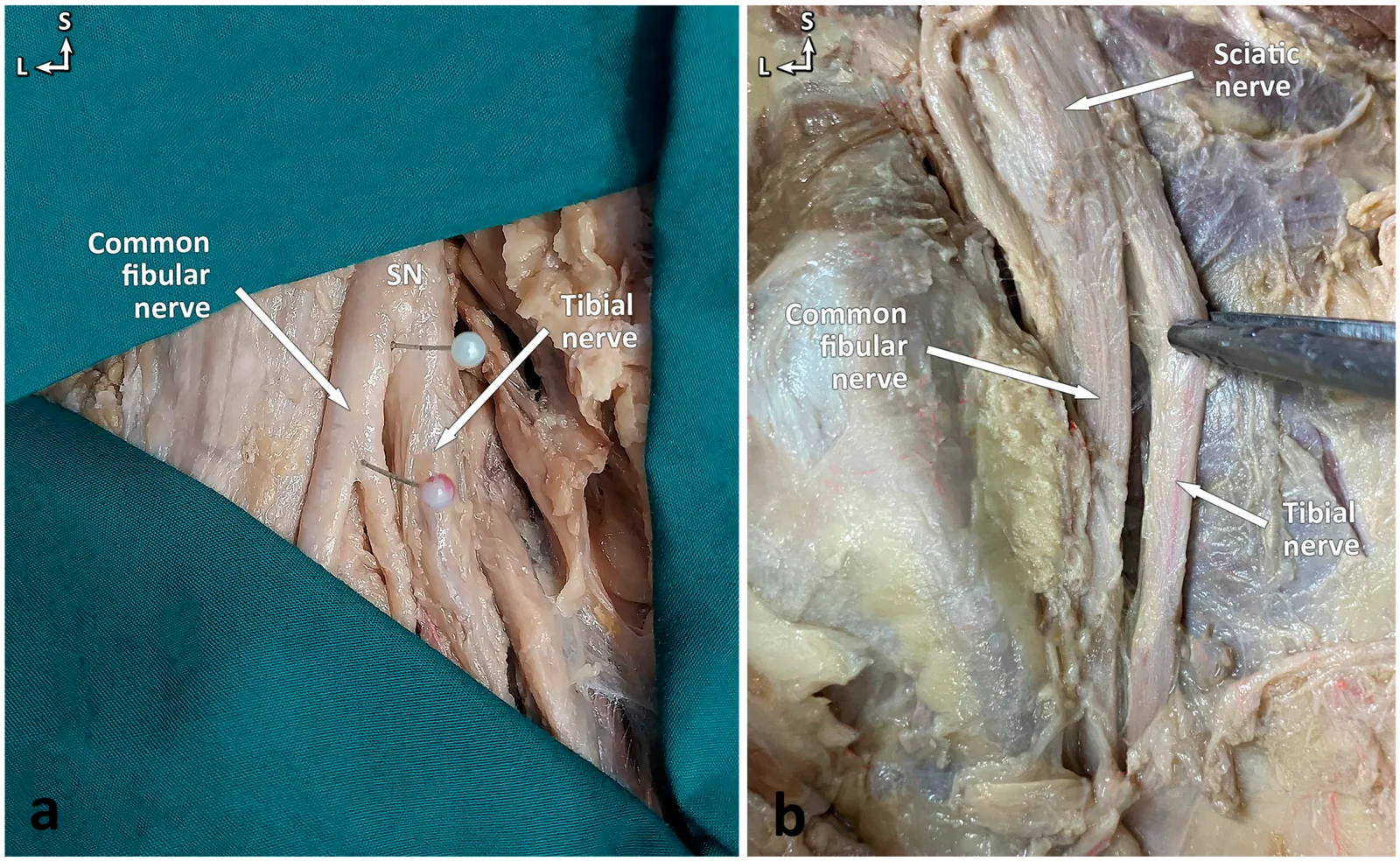
Representative dissection images showing different levels of sciatic nerve division. (**a**) Femoral-level bifurcation of the sciatic nerve in the posterior thigh. The sciatic nerve divides into the common fibular and tibial nerves distal to the gluteal region. (**b**) Gluteal-level division of the sciatic nerve, showing early separation of the common fibular and tibial components. S: superior; L: lateral; SN: sciatic nerve.

**Figure 3:**
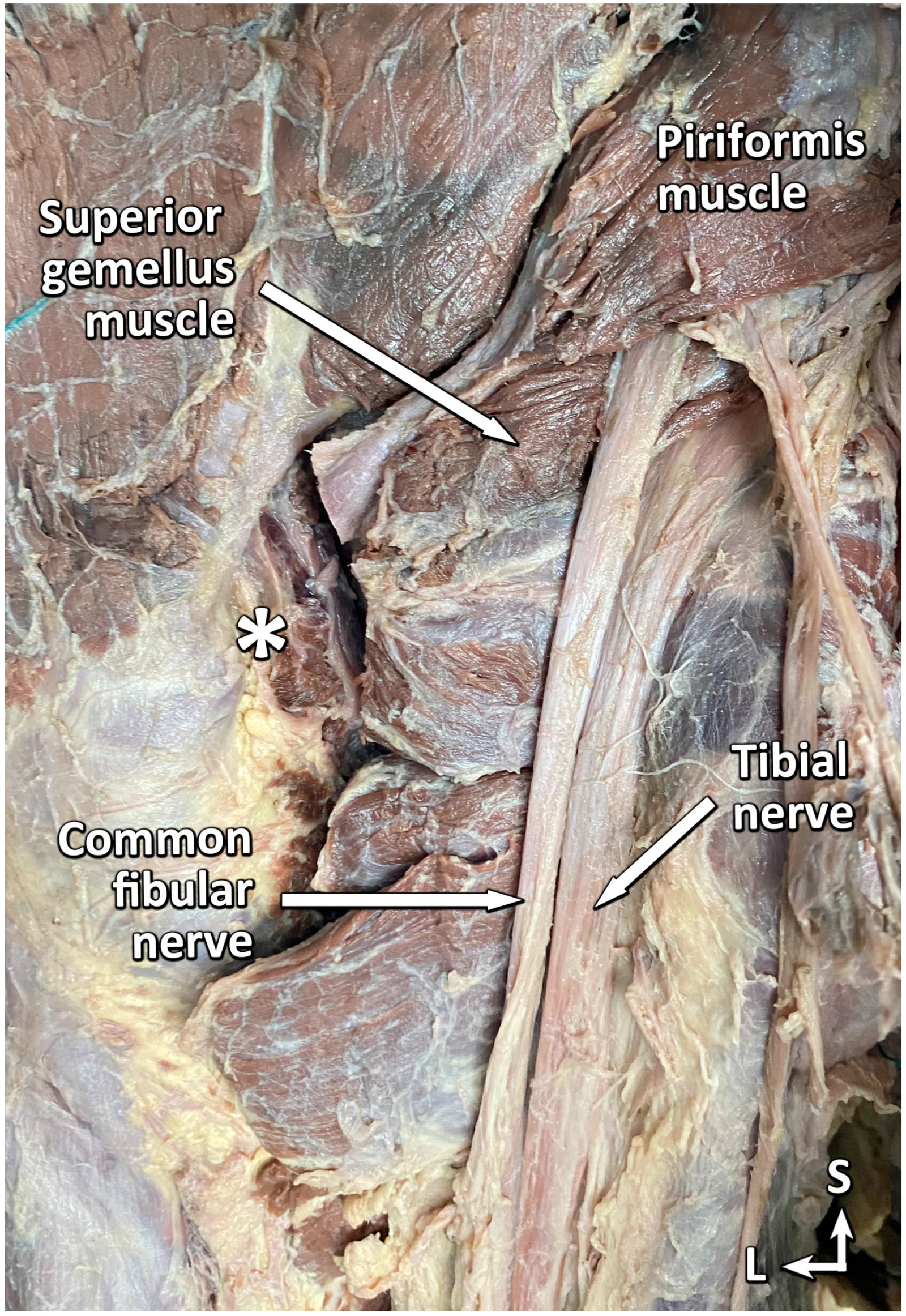
Separate emergence of the tibial and common fibular nerves associated with an atypical relationship to the superior gemellus muscle. The tibial and common fibular nerves course separately on opposite sides of the superior gemellus muscle in the deep gluteal region without forming a typical sciatic nerve trunk. This specimen was recorded as both a separate-emergence pattern and a superior gemellus-related atypical course. The asterisk indicates the greater trochanter. S: superior; L: lateral.

The bifurcation pattern was categorized in all 56 lower extremities based on its position relative to the inferior gluteal fold. Femoral-level bifurcation was the predominant pattern, observed in 51 specimens (91.1%) (Figure 2a). Gluteal-level bifurcation was observed in one specimen (1.8%) (Figure 2b). In four lower extremities (7.1%), the tibial and common fibular components emerged separately, without formation of a typical sciatic nerve trunk. One of these specimens also demonstrated an atypical superior gemellus-related course and is shown in Figure 3.

When bifurcation categories were evaluated by sex and side, the femoral-level bifurcation remained the most frequent pattern across all subgroups. The single gluteal-level bifurcation was observed in a right-sided female-derived specimen. Separate emergence of the tibial and common fibular components was observed in three male-derived and one female-derived lower extremity. The distribution of bifurcation categories according to sex and side is shown in Table 3.

In the dichotomized analysis, no significant association was observed between femoral versus non-femoral bifurcation and sex (*p* = 0.652) or side (*p* = 1.000).

### 3.2. Beaton and Anson Classification

According to the Beaton and Anson classification, Type A was the most common pattern, observed in 50 specimens (89.3%). Type B and Type D were each observed in two specimens (3.6%). In two additional specimens (3.6%), the neural components showed an atypical superior gemellus-related course that was not clearly represented in the classical Beaton and Anson classification; therefore, these cases were documented separately (Figure 3). The Beaton and Anson classification and the atypical superior gemellus-related pattern are summarized in Table 4.

When only the 54 specimens assignable to the classical Beaton and Anson classification were considered, Type A accounted for 50 specimens (92.6%), whereas Type B and Type D each accounted for two specimens (3.7%).

The distribution of Beaton and Anson patterns did not show a significant association with sex when dichotomized as Type A versus non-Type A (*p* = 0.652). Similarly, no significant association by side was observed (*p* = 0.682).

### 3.3. Morphometric Measurements

Morphometric measurements were analyzed based on the number of valid observations for each parameter. Specimens with separate emergence of the tibial and common fibular components were excluded from measurements requiring a typical sciatic nerve bifurcation point. Negative values for greater trochanter and ischial tuberosity measurements indicate that the bifurcation point was proximal to the corresponding reference level, whereas positive values indicate a distal location. Descriptive morphometric measurements are presented in Table 5. Although all 56 specimens were included in the anatomical classification, the number of valid observations varied among the morphometric parameters. The ASIS–LFC distance was available in 30 specimens, the GT and IT distances in 18 specimens each, and the PIRL distance in 44 specimens. Unavailable measurements resulted from insufficient preservation of the corresponding anatomical landmarks and, for measurements requiring a single bifurcation point, the separate-emergence pattern observed in four specimens.

Shapiro–Wilk testing indicated significant deviations from normality for the IT distance (W = 0.867, *p* = 0.016), PIRL distance (W = 0.681, *p* < 0.001), and IT ratio (W = 0.883, *p* = 0.029). In contrast, no significant deviations from normality were detected for the ASIS–LFC distance (W = 0.960, *p* = 0.301), GT distance (W = 0.932, *p* = 0.212), GT ratio (W = 0.929, *p* = 0.184), or PIRL ratio (W = 0.900, *p* = 0.059). Normality was also not consistently satisfied within the sex- and side-based subgroups.

The ASIS–LFC distance was available for 30 lower-extremity specimens. The mean distance was 44.61 ± 2.88 cm, with a median of 44.94 cm and a range of 39.00–49.50 cm.

The distance from the greater trochanter reference level to the sciatic nerve bifurcation point was measurable in 18 specimens. The mean signed distance was 23.13 ± 10.42 cm, with a median of 23.70 cm and a range of −4.50 to 40.00 cm. In one specimen, the bifurcation point was proximal to the greater trochanter reference level.

The distance from the ischial tuberosity reference level to the sciatic nerve bifurcation point was measurable in 18 specimens. The mean signed distance was 19.41 ± 9.10 cm, with a median of 22.60 cm and a range of −6.50 to 29.50 cm. As with the greater trochanter measurement, one specimen had a bifurcation point proximal to the ischial tuberosity reference level.

The distance between the posterior intercondylar reference line and the sciatic nerve bifurcation point was measurable in 44 specimens. All measurable bifurcation points were proximal to the posterior intercondylar reference line. The mean distance was 9.43 ± 6.97 cm, with a median of 7.00 cm and a range of 2.80–42.00 cm. The greatest distance, 42.00 cm, corresponded to the single gluteal-level bifurcation. When only femoral-level bifurcations were considered, the mean distance from the posterior intercondylar reference line was 8.67 ± 4.89 cm, with a median of 6.98 cm and a range of 2.80–22.00 cm.

### 3.4. Normalized Morphometric Ratios

Normalized morphometric ratios were calculated only for specimens in which both a valid ASIS–LFC distance and the corresponding landmark-to-bifurcation distance were available. The normalized ratio was calculated using the following formula:


Normalized ratio = landmark-to-bifurcation distance/ASIS–LFC distance × 100

The mean normalized greater trochanter ratio was 53.19 ± 23.46%, whereas the mean normalized ischial tuberosity ratio was 44.78 ± 20.73%. The mean normalized posterior intercondylar reference line ratio was 31.13 ± 20.20% across all specimens with the required measurements. When only femoral-level bifurcations were considered, the posterior intercondylar reference line-to-bifurcation distance corresponded to 27.82 ± 14.95% of the ASIS–LFC distance. Normalized morphometric ratios are provided in Table 6.

### 3.5. Sex- and Side-Based Morphometric Comparisons

Sex- and side-based comparisons were conducted using available valid measurements for each parameter. No statistically significant sex- or side-based differences were observed in ASIS–LFC distance, landmark-based bifurcation distances, or normalized ratios. Sex- and side-based morphometric comparisons are shown in Table 5.

## 4. Discussion

The present study evaluated the level of sciatic nerve bifurcation and its morphometric relationships with clinically relevant anatomical landmarks in 56 formalin-fixed adult lower extremities. Femoral-level bifurcation was the predominant pattern, separate emergence of the tibial and common fibular components was observed in a minority of specimens, and all measurable bifurcation points were located proximal to the posterior intercondylar reference line. Among femoral-level bifurcations, the mean distance from this line to the bifurcation point was 8.67 ± 4.89 cm. These findings demonstrate the longitudinal variability of sciatic nerve division and support the use of distal femoral landmarks as supplementary anatomical references during diagnostic imaging, ultrasound-guided procedures, and posterior thigh or popliteal interventions.

Although classical descriptions generally associate sciatic nerve division with the apex of the popliteal fossa, anatomical studies have documented bifurcation at variable levels extending from the gluteal region through the posterior thigh. Side-to-side variation within the same individual has also been reported [3,13,14]. The predominance of femoral-level bifurcation in the present study further supports the view that sciatic nerve division should be regarded as a variable anatomical event rather than a fixed feature of the popliteal region.

A clinically important aspect of the present study was the use of the posterior intercondylar reference line as a distal femoral landmark. All measurable bifurcation points were proximal to this line, and restricting the analysis to femoral-level bifurcations reduced the influence of the single gluteal-level outlier. Distal femoral bony landmarks may provide more reproducible anatomical orientation than soft-tissue landmarks such as the popliteal crease, which can vary with knee position, body habitus, skin folding, and soft-tissue thickness. Barut et al. similarly reported that the sciatic nerve bifurcated proximal to the transverse plane of the distal femoral condyles and suggested that the condyles may provide a more reliable reference than the popliteal crease [10]. The present findings extend this concept by defining the relationship of the bifurcation point to a transverse line connecting the posterior aspects of the femoral condyles.

From a diagnostic and ultrasound-guided procedural perspective, the posterior intercondylar reference line should not be used as a substitute for direct visualization of the sciatic nerve and its terminal branches. Rather, it may serve as an anatomical reference to guide the operator in determining where to begin scanning or where the bifurcation zone may be expected relative to the posterior knee region. Previous ultrasonographic studies have shown that the sciatic nerve and its terminal components can be visualized in the popliteal region. Ricci reported that the sciatic nerve and its tibial and common fibular branches were visible using duplex ultrasonography in all examined lower limbs and highlighted the importance of nerve identification for diagnostic and interventional procedures in the popliteal fossa [5]. Chen et al. provided ultrasonographic reference values for the sciatic nerve in healthy volunteers and demonstrated that high-resolution ultrasonography can be used to assess sciatic nerve morphology along its course [4]. Singh et al. reported that the sciatic nerve terminated approximately 77 mm proximal to the popliteal crease and emphasized the clinical value of sonographic assessment of the bifurcation level and nerve depth during popliteal procedures [11]. These findings support the diagnostic value of combining knowledge of anatomical landmarks with real-time ultrasonographic assessment.

The present morphometric findings also align with previous ultrasound-guided anesthesia studies, which report that the sciatic nerve bifurcation is commonly located several centimeters proximal to the popliteal crease. Gürkan et al. observed the sciatic nerve bifurcation at a mean distance of 6.9 ± 1.6 cm proximal to the popliteal crease and showed that patient positioning may improve ultrasonographic visibility of the nerve in the popliteal region [15]. Vrzgula et al. reported that the bifurcation occurred at a mean distance of 68.1 ± 19.3 mm proximal to the popliteal crease and emphasized that an injection distal to the bifurcation may affect only one terminal component, potentially resulting in an incomplete blockade [16]. Griseto et al. similarly noted that the bifurcation is often found 5–10 cm proximal to the popliteal crease and recommended dynamic ultrasound scanning to differentiate the tibial and common fibular nerves at the bifurcation [9]. The mean femoral-level distance observed in the present study, 8.67 cm proximal to the posterior intercondylar reference line, is broadly consistent with clinical and ultrasound-based observations. However, because the present study was conducted on formalin-fixed specimens and lacked real-time ultrasonographic validation, this comparison should be interpreted as anatomical concordance rather than procedural proof.

The clinical relevance of the sciatic nerve bifurcation level is particularly evident in the popliteal sciatic nerve block. Deposition of local anesthetic distal to the bifurcation may predominantly affect only one terminal component and result in an incomplete sensory or motor block. The variable separation of the tibial and common fibular components and their relationship within the common epineural sheath provide the anatomical basis for this concern [7,17]. Double-stimulation or double-injection techniques and approaches targeting the nerves at or distal to their separation have been reported to influence block onset or completeness [18,19,20,21], while catheter position relative to the bifurcation may affect postoperative analgesia during continuous popliteal sciatic nerve block [22]. Knowledge of the expected bifurcation zone may therefore assist procedural planning, although real-time ultrasonographic confirmation remains essential.

Another important finding was the separate emergence of the tibial and common fibular components without formation of a typical sciatic nerve trunk in 7.1% of lower extremities. Comparable patterns in which the two components remain separate throughout their course have been documented in previous anatomical studies [3,23]. Because definitions and classification systems differ among studies, direct prevalence comparisons should be made cautiously. Clinically, this configuration challenges the assumption that a single sciatic nerve trunk will always be identifiable in the posterior thigh. Ultrasound-guided examination should therefore confirm whether the two components form a common trunk or remain separate before an injection or intervention is planned.

The Beaton and Anson classification remains the most widely used system for describing the relationship between the sciatic nerve and the piriformis muscle. In the present study, Type A was the predominant Beaton and Anson pattern, whereas Types B and D were uncommon. This distribution is consistent with the original Beaton and Anson classification and subsequent cadaveric studies showing that passage of an undivided sciatic nerve inferior to the piriformis muscle is the most frequent configuration, although reported prevalences vary among populations [12,24,25]. Recognition of non-classical relationships remains important during posterior hip surgery, deep gluteal procedures, proximal sciatic nerve block, and assessment of sciatic nerve entrapment.

The superior gemellus-related course observed in two specimens could not be adequately represented by the classical piriformis-based Beaton and Anson classification. In these specimens, the tibial and common fibular components coursed on opposite sides of the superior gemellus muscle. This observation should not be interpreted as establishing a new classification type; rather, it illustrates that clinically relevant sciatic nerve variation may also involve the short external rotator muscles. The sciatic nerve is closely related to these structures in the deep gluteal space, and their anatomical relationships may influence ultrasound-guided localization and the assessment of deep gluteal syndrome or nerve entrapment [9,26,27].

High-resolution ultrasonography is also used to assess peripheral nerve morphology, entrapment, traumatic lesions, and postoperative or iatrogenic injury. Reference data and knowledge of expected anatomical variation are therefore important for distinguishing normal configurations from pathological findings. Chen et al. demonstrated that sciatic nerve morphology may vary with anthropometric characteristics and sex [4]. No statistically significant sex- or side-based differences were identified in the present study; however, these comparisons were limited by parameter-specific missing measurements and should be considered descriptive rather than definitive evidence of symmetry or the absence of sexual dimorphism.

The greater trochanter and ischial tuberosity provide complementary proximal landmarks, particularly during subgluteal localization of the sciatic nerve. Franco highlighted the practical value of surface anatomical landmarks during posterior approaches to the sciatic nerve [28]. Although measurements based on the greater trochanter and ischial tuberosity were available in a smaller subset of specimens, their integration with the posterior intercondylar reference line may help define a broader proximal-to-distal anatomical corridor for tracing the nerve. Such landmark-based estimates should remain supportive rather than definitive, particularly in individuals with obesity, altered anatomy, postoperative changes, or poor sonographic windows.

Overall, these findings underscore the importance of considering the sciatic nerve bifurcation as an anatomical variable and suggest that distal femoral and popliteal landmarks may provide useful orientation for diagnostic imaging, regional anesthesia, and posterior-thigh or popliteal surgical procedures.

### Limitations and Future Directions

The findings of the present study should be interpreted in light of its cadaveric design and specimen-related constraints. Formalin fixation may have altered tissue dimensions, compliance, and spatial relationships compared with living anatomy. Fixation and specimen positioning may also distort soft-tissue landmarks, particularly the inferior gluteal fold used to distinguish gluteal- from femoral-level bifurcations; therefore, classifications close to this boundary should be interpreted cautiously. Incomplete preservation of some anatomical landmarks resulted in parameter-specific missing measurements, particularly for the greater trochanter and ischial tuberosity. Consequently, these proximal-landmark findings should be considered descriptive and interpreted with caution. The anatomical collection comprised both bilateral pairs and isolated unilateral specimens; complete donor-matched bilateral data were therefore unavailable, precluding systematic within-donor right–left comparisons. Only the adult status of the donors was known, as detailed age and anthropometric data were unavailable. The limited number of uncommon anatomical variants also restricted robust prevalence estimates and subgroup comparisons. In addition, each morphometric parameter was measured once by two observers working together and recorded by consensus; because independent repeated measurements were not performed, formal intraobserver and interobserver reliability could not be assessed. No direct in vivo ultrasonographic validation was performed. Accordingly, the reported measurements should be regarded as anatomical reference data rather than direct clinical or procedural thresholds.

Future studies should include larger samples of donor-matched bilateral specimens, standardized preservation protocols, complete preservation of the relevant landmarks, and detailed demographic and anthropometric data. Repeated independent measurements would enable formal assessment of intraobserver and interobserver reliability. Combining cadaveric morphometry with real-time ultrasonographic assessment in living individuals would further clarify the clinical applicability of the posterior intercondylar reference line and the other anatomical landmarks evaluated in this study.

## 5. Conclusions

This cadaveric morphometric study found that sciatic nerve bifurcation most commonly occurred at the femoral level, with gluteal-level division and separate emergence of the tibial and common fibular components also observed. All measurable bifurcation points were proximal to the posterior intercondylar reference line, suggesting that this distal femoral reference may serve as a practical anatomical landmark for orientation in the posterior knee and distal thigh. The findings may be relevant to diagnostic ultrasonography, ultrasound-guided popliteal sciatic nerve block, posterior-thigh interventions, and surgical approaches involving the sciatic nerve. However, the posterior intercondylar reference line should not be considered a substitute for direct visualization of the sciatic nerve and its terminal branches.

## Figures and Tables

**Table 1:** Summary of the anatomical reference points and morphometric measurements.

Parameter	Definition and Reference Points	Recording and Interpretation
**Greater trochanter distance**	Straight-line distance between the sciatic nerve bifurcation point and the reference level of the most prominent point of the greater trochanter	Recorded as a signed value; negative values indicate a proximal and positive values a distal bifurcation
**Ischial tuberosity distance**	Straight-line distance between the sciatic nerve bifurcation point and the reference level of the most prominent point of the ischial tuberosity	Recorded as a signed value; negative values indicate a proximal and positive values a distal bifurcation
**Posterior intercondylar reference line distance**	Straight-line distance from the transverse line connecting the posterior aspects of the medial and lateral femoral condyles to the sciatic nerve bifurcation point	Recorded as the proximal distance from the reference line to the bifurcation point
**Anterior superior iliac spine–lateral femoral condyle reference distance**	Straight-line distance between the anterior superior iliac spine and the lateral femoral condyle	Used as the lower-extremity size reference for normalization
**Normalized ratio**	Landmark-to-bifurcation distance divided by the anterior superior iliac spine–lateral femoral condyle reference distance × 100	Expressed as a percentage

**Table 2:** General specimen characteristics and bifurcation-level distribution.

Variable	Category	*n*	%
Sex	Male-derived	38	67.9
	Female-derived	18	32.1
Side	Right	31	55.4
	Left	25	44.6
Bifurcation category	Femoral-level bifurcation	51	91.1
	Gluteal-level bifurcation	1	1.8
	Separate emergence	4	7.1
Total		56	100

**Table 3:** Distribution of bifurcation categories according to sex and side.

Bifurcation Category	Sex		Side	
	Male-Derived (*n* = 38)	Female-Derived (*n* = 18)	Right (*n* = 31)	Left (*n* = 25)
Femoral-level bifurcation	35 (%92.1)	16 (%88.9)	28 (%90.3)	23 (%92.0)
Gluteal-level bifurcation	0 (%0)	1 (%5.6)	1 (%3.2)	0 (%0)
Separate emergence	3 (%7.9)	1 (%5.6)	2 (%6.5)	2 (%8.0)

Data are presented as *n* (%). Percentages were calculated within each sex and side category.

**Table 4:** Beaton and Anson (BA) classification and atypical superior gemellus (SG)-related pattern.

Pattern	*n*	% of All Specimens	% of BA-Assignable Specimens
Type A	50	89.3	92.6
Type B	2	3.6	3.7
Type D	2	3.6	3.7
SG-related atypical pattern	2	3.6	Not applicable
Total	56	100	100

**Table 5:** Overall, sex-based, and side-based morphometric data.

Parameter	Overall (Valid *n*; Mean ± SD)	Male (*n*; Mean ± SD)	Female (*n*; Mean ± SD)	*p* Value	Right (n; Mean ± SD)	Left (*n*; Mean ± SD)	*p* Value
**ASIS–LFC distance, cm**	30; 44.61 ± 2.88	20; 44.77 ± 2.72	10; 44.29 ± 3.32	0.965	16; 44.33 ± 2.81	14; 44.92 ± 3.04	0.546
**GT distance, signed, cm**	18; 23.13 ± 10.42	10; 24.08 ± 10.10	8; 21.94 ± 11.38	0.859	9; 19.47 ± 11.93	9; 26.79 ± 7.63	0.233
**IT distance, signed, cm**	18; 19.41 ± 9.10	10; 19.51 ± 7.68	8; 19.28 ± 11.19	0.894	9; 17.24 ± 10.89	9; 21.57 ± 6.85	0.377
**PIRL distance, all measurable cases, cm**	44; 9.43 ± 6.97	28; 8.39 ± 5.29	16; 11.24 ± 9.14	0.184	24; 9.77 ± 8.07	20; 9.02 ± 5.56	0.680
**GT ratio, %**	18; 53.19 ± 23.46	10; 54.43 ± 21.32	8; 51.64 ± 27.32	0.897	9; 45.18 ± 26.36	9; 61.20 ± 18.16	0.251
**IT ratio, %**	18; 44.78 ± 20.73	10; 44.39 ± 16.85	8; 45.25 ± 26.05	0.762	9; 40.05 ± 23.92	9; 49.50 ± 17.07	0.377
**PIRL ratio, all measurable paired cases, %**	18; 31.13 ± 20.20	10; 27.45 ± 18.09	8; 35.74 ± 22.96	0.633	9; 34.77 ± 24.49	9; 27.50 ± 15.42	0.724

Values are presented as valid *n*; mean ± standard deviation. Sex- and side-based comparisons were performed using the Mann–Whitney U test. The overall values are descriptive and were not included in the inferential comparisons. Negative signed values indicate that the bifurcation point was proximal to the corresponding reference level, whereas positive values indicate a distal location. Normalized ratios were calculated as the corresponding landmark-to-bifurcation distance divided by the ASIS–LFC distance × 100. Abbreviations: ASIS, anterior superior iliac spine; LFC, lateral femoral condyle; GT, greater trochanter; IT, ischial tuberosity; PIRL, posterior intercondylar reference line; SD, standard deviation.

**Table 6:** Normalized morphometric ratios.

Parameter	Valid *n*	Mean ± SD, %	Median, %	Min–Max, %	IQR, %
GT distance/ASIS–LFC distance × 100	18	53.19 ± 23.46	51.81	−9.38–86.96	46.09–70.06
IT distance/ASIS–LFC distance × 100	18	44.78 ± 20.73	48.88	−13.54–70.91	37.32–58.82
PIRL distance/ASIS–LFC distance × 100, all measurable cases	18	31.13 ± 20.20	29.24	5.96–87.50	13.72–43.69
PIRL distance/ASIS–LFC distance × 100, femoral-level bifurcations only	17	27.82 ± 14.95	27.65	5.96–49.77	10.82–41.43

Abbreviations: GT, greater trochanter; IT, ischial tuberosity; PIRL, posterior intercondylar reference line; ASIS–LFC, anterior superior iliac spine–lateral femoral condyle; IQR, interquartile range.

## Data Availability

The primary data supporting the findings of this study are included within the article. Additional anonymized data may be obtained from the corresponding author upon reasonable request.

## Abbreviations

The following abbreviations are used in this manuscript:

SN	Sciatic nerve
SG	Superior gemellus
BA	Beaton and Anson
GT	Greater trochanter
IT	Ischial tuberosity
PIRL	Posterior intercondylar reference line
SD	Standard deviation
IQR	Interquartile range
ASIS–LFC	Anterior superior iliac spine–lateral femoral condyle
